# Screening of Mild Cognitive Impairment Through Conversations With Humanoid Robots: Exploratory Pilot Study

**DOI:** 10.2196/42792

**Published:** 2023-01-13

**Authors:** Kenta Yoshii, Daiki Kimura, Akihiro Kosugi, Kaoru Shinkawa, Toshiro Takase, Masatomo Kobayashi, Yasunori Yamada, Miyuki Nemoto, Ryohei Watanabe, Miho Ota, Shinji Higashi, Kiyotaka Nemoto, Tetsuaki Arai, Masafumi Nishimura

**Affiliations:** 1 Department of Informatics Graduate School of Intergraded Science and Technology Shizuoka University Hamamatsu Japan; 2 Neuro-Symbolic AI IBM Research Tokyo Japan; 3 Accessibility Research IBM Research Tokyo Japan; 4 Digital Health IBM Research Tokyo Japan; 5 Healthcare and Life Science IBM Consulting IBM Japan, Ltd Tokyo Japan; 6 Department of Psychiatry Division of Clinical Medicine Faculty of Medicine, University of Tsukuba Ibaraki Japan; 7 Department of Psychiatry Ibaraki Medical Center Tokyo Medical University Ibaraki Japan

**Keywords:** mild cognitive impairment, Alzheimer disease, neuropsychiatric symptoms, neuropsychological assessment, simple screening, humanoid robot, robot, symptoms, neuropsychological, monitoring

## Abstract

**Background:**

The rising number of patients with dementia has become a serious social problem worldwide. To help detect dementia at an early stage, many studies have been conducted to detect signs of cognitive decline by prosodic and acoustic features. However, many of these methods are not suitable for everyday use as they focus on cognitive function or conversational speech during the examinations. In contrast, conversational humanoid robots are expected to be used in the care of older people to help reduce the work of care and monitoring through interaction.

**Objective:**

This study focuses on early detection of mild cognitive impairment (MCI) through conversations between patients and humanoid robots without a specific examination, such as neuropsychological examination.

**Methods:**

This was an exploratory study involving patients with MCI and cognitively normal (CN) older people. We collected the conversation data during neuropsychological examination (Mini-Mental State Examination [MMSE]) and everyday conversation between a humanoid robot and 94 participants (n=47, 50%, patients with MCI and n=47, 50%, CN older people). We extracted 17 types of prosodic and acoustic features, such as the duration of response time and jitter, from these conversations. We conducted a statistical significance test for each feature to clarify the speech features that are useful when classifying people into CN people and patients with MCI. Furthermore, we conducted an automatic classification experiment using a support vector machine (SVM) to verify whether it is possible to automatically classify these 2 groups by the features identified in the statistical significance test.

**Results:**

We obtained significant differences in 5 (29%) of 17 types of features obtained from the MMSE conversational speech. The duration of response time, the duration of silent periods, and the proportion of silent periods showed a significant difference (*P*<.001) and met the reference value r=0.1 (small) of the effect size. Additionally, filler periods (*P*<.01) and the proportion of fillers (*P*=.02) showed a significant difference; however, these did not meet the reference value of the effect size. In contrast, we obtained significant differences in 16 (94%) of 17 types of features obtained from the everyday conversations with the humanoid robot. The duration of response time, the duration of speech periods, jitter (local, relative average perturbation [rap], 5-point period perturbation quotient [ppq5], difference of difference of periods [ddp]), shimmer (local, amplitude perturbation quotient [apq]3, apq5, apq11, average absolute differences between the amplitudes of consecutive periods [dda]), and F0cov (coefficient of variation of the fundamental frequency) showed a significant difference (*P*<.001). In addition, the duration of response time, the duration of silent periods, the filler period, and the proportion of fillers showed significant differences (*P*<.05). However, only jitter (local) met the reference value r=0.1 (small) of the effect size. In the automatic classification experiment for the classification of participants into CN and MCI groups, the results showed 66.0% accuracy in the MMSE conversational speech and 68.1% accuracy in everyday conversations with the humanoid robot.

**Conclusions:**

This study shows the possibility of early and simple screening for patients with MCI using prosodic and acoustic features from everyday conversations with a humanoid robot with the same level of accuracy as the MMSE.

## Introduction

### Background

The rising number of patients with dementia has become a serious social problem worldwide. According to the World Health Organization (WHO), the number of patients with dementia will reach 75 million by 2030 [1]. As the number of patients with dementia increases, its cost, such as nursing care, is estimated to rise to US $2 trillion, and there are concerns that this will lead to social and economic losses [1]. There is an urgent need to counteract the increase in patients with dementia.

Correct diagnosis and starting treatment can cure some types of dementia. However, we have not found any breakthrough treatments to date for Alzheimer disease and Lewy body dementia, which account for most of the cases. We can only delay the progress and improve the symptoms with medication.

Dementia is diagnosed comprehensively using cognitive function tests, such as the revised Hasegawa method simple intelligence assessment scale (HDS-R) [2] and the Mini-Mental State Examination (MMSE) [3], and MRI. These tests are not easily accessible to patients because not only do they need the patient to visit a hospital or a clinic but also the shortage of doctors, the time and cost, and the mental and physical stress caused by cognitive function tests and MRI can deter them.

We need a simple dementia-screening method that is less burdensome for the patient and routinely available for early detection of dementia, especially for patients with mild cognitive impairment (MCI) [4] at the precursor stage. There have been numerous studies in this field: some have examined the efficacy of testing using devices, such as tablets [5-7] and virtual reality [8-10], rather than the tests performed by a doctor as a simple method mentioned earlier, and others have tried to find signs of dementia and MCI from conversational speech [11-22], although many of these methods are not suitable for everyday use as they are tests for cognitive function or conversational speech. In contrast, we expect to save labor in nursing care facilities that have a severe problem with personnel shortages using a robot that can monitor and talk to older people [23-25]. If we can find signs of cognitive decline from everyday conversations between a robot and older people, it may help us detect early stages of dementia without conducting a particular test, while letting the older people enjoy conversations.

### Objectives

This study focuses on the early detection of MCI through conversations between patients and humanoid robots without a specific examination, such as neuropsychological examination. We analyzed speech features from the recorded everyday conversations between participants (cognitively normal [CN] people and patients with MCI) and humanoid robots to identify the effective features for detecting the signs of cognitive decline. We also conducted an automatic classification experiment with patients with MCI and CN older people using the identified features and examined the possibility of a simple dementia-screening test using their everyday conversations with humanoid robots.

### Related Studies

#### Attempts To Detect Dementia Early Using Conversational Speech

Kato et al [11] extracted 128 types of prosodic features, such as fundamental frequency, formant, and Mel-frequency cepstral coefficient (MFCC), from the questions included in the HDS-R. Their study revealed that it is possible to assess cognitive impairment from speech based on the correlation between the feature selected by the feature selection method and the HDS-R score. Furthermore, their classification experiment using logistic regression revealed that it could classify participants into CN people and patients with dementia, and CN people and patients with MCI, with 89.5% and 75.9% accuracy, respectively. Roark et al [12] conducted a statistical significance test by extracting verbal and acoustic features from speech and transcriptions during multiple cognitive function tests, such as the MMSE. Their study showed significant differences in more than 1 feature between CN older people and patients with MCI, such as phonation time. They also showed from a classification experiment using a support vector machine (SVM) that the accuracy would improve more when classifying patients by adding the values of verbal and acoustic features than by the scores of cognitive function tests only.

However, we know that major depressive disorder can also reduce cognitive function, and depression can occur as a peripheral symptom of dementia. It is necessary to identify whether a patient's cognitive decline is due to dementia or major depressive disorder. Thus, Sumali et al [13] conducted an automatic classification experiment using statistical significance tests and linear kernels of an SVM by recording 10-minute clinical interviews doctors conducted with patients with major depressive disorder and dementia and their 20-minute audio during the major depressive disorder and dementia tests. The result showed a significant difference between patients with depression and those with dementia. Furthermore, it showed the possibility of classifying cognitive decline into the types prompted by major depressive disorder and by dementia.

These studies showed the effectiveness of using the values of linguistic and acoustic features obtained from conversational speech. However, many of these studies used cognitive function tests as the subject of their analyses. It is not easy to conduct cognitive function tests routinely, since we need specialized doctors and psychologists to do it. In addition, they are not suitable for everyday use as patients may remember the questions and their replies if the tests are conducted multiple times, which leads to inaccurate results. Therefore, Ali et al [15] focused on the spontaneous utterances of older people rather than structured conversations that generally have fixed answers to questions, such as cognitive function tests. They extracted 20 prosodic features and 18 linguistic features and conducted separate statistical significance tests by age, gender, and educational level. The results showed that there were significant differences between CN people and those with Alzheimer disease in multiple feature values, such as the response time and the proportion of the duration of silent periods in speech. Moreover, their classification experiments using an SVM, etc, revealed that it is possible to classify patients into CN people and those with Alzheimer disease with up to 86% accuracy, showing the possibility of identifying patients with Alzheimer disease from spontaneous speech. Tóth et al [17] reported that they obtained significant differences in the duration of the speech period, the proportions of silent periods, and fillers (words that fill the silence, such as “hmm” and “ah”) in speech when they analyzed the spontaneous utterances of participants comprising CN people and patients with MCI (1) immediately after showing them a minute-long video and (2) a while after showing them the video to discuss it again. These results showed that it is possible to identify patients with dementia and patients with MCI from spontaneous speech rather than structured speech, such as that used in cognitive function tests. However, these previous studies collected data manually, which makes them difficult to be computerized for future use and unsuitable for medical and nursing care facilities where the problem of personnel shortages is acute.

#### Attempts for the Early Detection of Dementia Using Conversational Speech With a Dialogue System

There have been attempts to collect conversational speeches using a dialogue system instead of doing so manually using human labor to help doctors detect dementia early. Tanaka et al [19] conducted a statistical significance test by extracting the values of linguistic and acoustic features from participants' conversations with a virtual agent on computers. The result showed differences between CN people and patients with dementia in multiple features, such as response time and coefficient of variation of *F*_0_. Hall et al [21] extracted acoustic features, such as jitter and shimmer, from the conversations of healthy controls, patients with MCI, and patients with Alzheimer disease with a dialogue system on a tablet and performed a statistical significance test. The result showed that there was a significant difference between the 3 groups in the features, such as jitter (local), shimmer (local), and shimmer (apq3). Their classification experiment using an SVM showed that it was possible to classify the participants into healthy controls and patients with Alzheimer disease with an accuracy of up to 92.6% and healthy controls and patients with MCI with an accuracy of up to 84.4%. These studies have shown that a dialogue system can identify patients with Alzheimer disease and patients with MCI as cognitive function tests and conversations with a person do. However, these dialogues are with virtual existences on computers and tablets.

We hope that the use of robots will save labor and help detect early signs of dementia in medical and nursing care facilities where the problem of labor shortages is severe. Jonell et al [26] have been attempting to identify the signs of dementia from the results of cognitive function tests performed by robots in a project called EACare. In their attempt for the early identification of patients with MCI from the results of cognitive function tests performed by robots installed in nursing homes, Luperto et al [27] assessed whether older people would accept the use of robots. Their analysis showed that older people approve of robots that provide explanations and supervise cognitive function tests instead of clinicians. However, these studies concern cognitive function tests, and few studies have analyzed speech features in chats between older people and physically existing robots.

## Methods

### Study Design

This research was an exploratory study involving patients with MCI and CN older people.

We recorded everyday conversations between the participants (CN older people, patients with MCI) and humanoid robots, in addition to conversations during the MMSE. We conducted statistical analyses of speech features from these conversations to clarify the speech features of patients with MCI based on the doctors' diagnostic classification into CN and MCI from various tests, such as cognitive function tests, blood tests, and MRI.

We conducted an automatic classification experiment using these features and examined the possibility of a simple and easy screening test for dementia using people's everyday conversations with humanoid robots.

### Recruitment

We collected the conversation data of a total of 94 participants. Of these, 47 (50%) patients had MCI and 47 (50%) were CN older people. We used the same collection method as that in the previous study [28]. For participants in the experiment, we recruited patients with MCI from among outpatients at the Department of Psychiatry, University of Tsukuba Hospital, and CN older people from among patients' spouses or through employment agencies and local ads inside Ibaraki Prefecture. All of them were Japanese speakers.

Two psychiatrists (authors TA and KN), who are experts in dementia, confirmed the patients' diagnoses from their clinical records, cognitive function tests, and MRI tests regardless of the diagnoses they had received before participating in this study.

As mentioned earlier, there are various types of dementia, such as Alzheimer disease and frontotemporal lobar degeneration. Busse et al [29] reported that MCI can also be divided into 4 subtypes. However, we treated the participants as patients with MCI without dividing them into those categories in this study.

#### Data Exclusion

We excluded people with severe mental illnesses (major depression, bipolar disorder, schizophrenia) or difficulty speaking Japanese.

### Ethical Considerations

The study was conducted with the approval of the Ethics Committee, University of Tsukuba Hospital (H29-065), and it followed the ethical code for research with humans, as stated in the Declaration of Helsinki. All participants provided written informed consent to participate in the study. The collected data were anonymized so that individuals could not be identified and used for analysis.

The reward for participating in the experiment was JPY 3000 (US $22.01) for patients with MCI, while CN older people participated as volunteers.

### Collection of Conversation Data

#### Cognitive Function Tests

A specialized psychologist recorded Japanese conversational speech when conducting cognitive function tests on the participants, such as the MMSE [3] and the Frontal Assessment Battery (FAB) [30], to examine the function of the frontal lobe. In this study, we focused on conversational speech when we conducted the MMSE, which is the test previous studies have also used. The MMSE consists of 11 questions, including oral questions on perception and memory and questions that involve writing/drawing, such as copying a figure.

This study tested temporal orientation and spatial orientation for the analysis. To test temporal orientation, we asked the date, day, and season (4 seasons) on the day of the experiment, and to test spatial orientation, we asked the name of the prefecture, city, region, name of the building (or type), and the number of floors where we were experimenting. The participants provided oral answers in both tasks, and we assessed them on a 5-point scale. Table 1 shows the participants' attributes and average scores on temporal and spatial orientations.

**Table 1:** Demographic and clinical characteristics of study participants (N=94).

Characteristics			Participants	
		CN^a^ (n=47, 50%)		MCI^b^ (n=47, 50%)
**Gender, n (%)**				
	Female	27 (57)		20 (43)
**Age (years)**				
	Mean (SD)	70.6 (4.9)		74.0 (5.4)
	Range	61-80		61-87
**Temporal orientation score on the MMSE^c^**				
	Mean (SD)	4.6 (0.6)		4.5 (0.7)
	Range	3-5		2-5
**Special orientation score on the MMSE**				
	Mean (SD)	4.7 (0.5)		4.7 (0.5)
	Range	3-5		4-5

^a^CN: cognitively normal.

^b^MCI: mild cognitive impairment.

^c^MMSE: Mini-Mental State Examination.

#### Everyday Conversations With a Humanoid Robot

We recorded the participants' everyday conversational speech with a humanoid robot Pepper [30], developed by SoftBank Robotics in 2014. Pepper can communicate through voice and a tablet in the chest. Pepper's movements and conversations are easily programmable and have been used in health care, nursing, and educational facilities.

**Figure 1:**
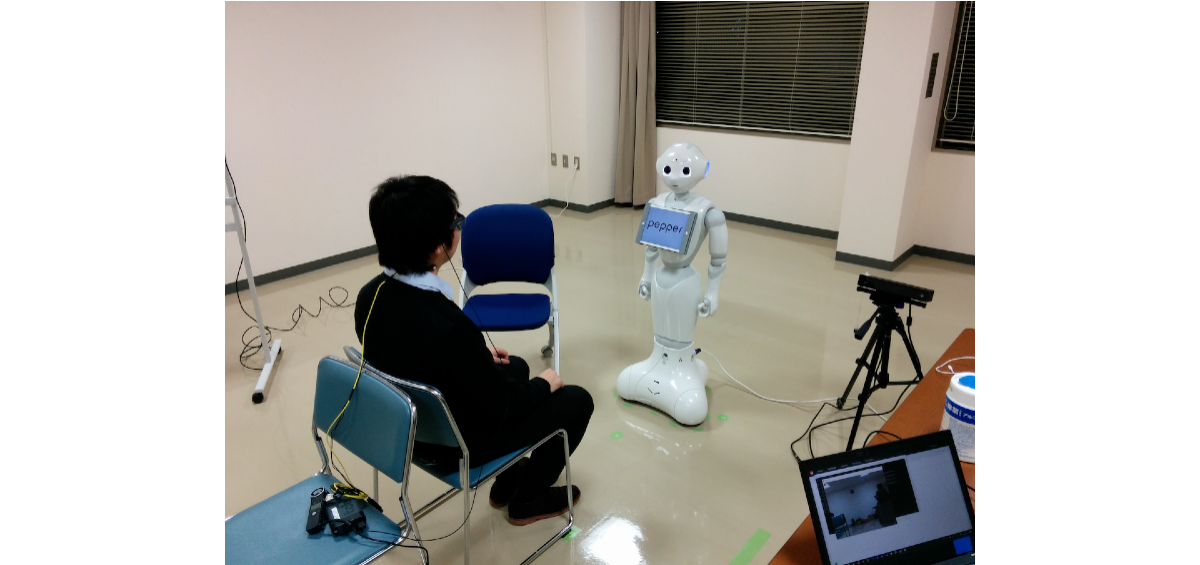
Participant in conversation with a humanoid robot.

Figure 1 shows how we recorded the conversational data between the participants and the robot. We conducted a Wizard of Oz (WoZ) experiment. The operator sat in a position that kept them out of sight of the participants. They switched topics or selected the content of conversations according to the participants’ replies while following the basic scenario prepared beforehand.

Although we recorded the conversational data in WoZ format in this study, the design of the scenario assumed a 1-question-1-answer type of dialogue that we considered feasible enough in the current automatic dialogue system, rather than free dialogue like the kind between humans. In addition, we developed the scenario after conducting several trial experiments on the CN older people. The scenario is shown in Textbox 1.

**The scenario of the conversation with a humanoid robot Pepper.**

**Step 1: Introduction**

A total of 3 questions, including Pepper's self-introduction, whether they had known Pepper before, and their first impression of Pepper

**Step 2: Health**

A total of 4 questions, including their physical condition on the present day, whether they were able to sleep the previous night, and things they are mindful about to stay healthy

**Step 3: Diet**

A total of 5 questions, including the meals they had the previous day, how to prepare those meals, and what they like about their favorite food

**Step 4: Conclusion**

Thanking them for the conversation and some utterances to signal the end of the experiment

The scenario consists of a question-reply section and a chat section. In the question-reply section, we inserted questions that test memory and the ability to think logically that have the potential to identify a decline in cognitive function (eg, details of the meals the participants had the previous night and how to prepare them). Meanwhile, the system delivers appropriate replies and self-disclosure of information according to the participants' responses to simulate everyday conversations in the chat section. The WoZ operator set Pepper's replies by judging whether the content of the participants' responses was positive, negative, or neither. For example, Pepper's reply when participants say they know about the robot is “Wow! I'm glad” and when they say they do not know about the robot is “Mmm, that’s a shame.”

To create an environment close to everyday conversation, we did not give the participants instructions in advance that would narrow down their choice of comments, such as “Do not ask the robot questions.” When the participants asked the robot questions, the operator avoided them by replying, “I can't answer that question,” or moved on to the next topic.

#### Recording Equipment

**Figure 2:**
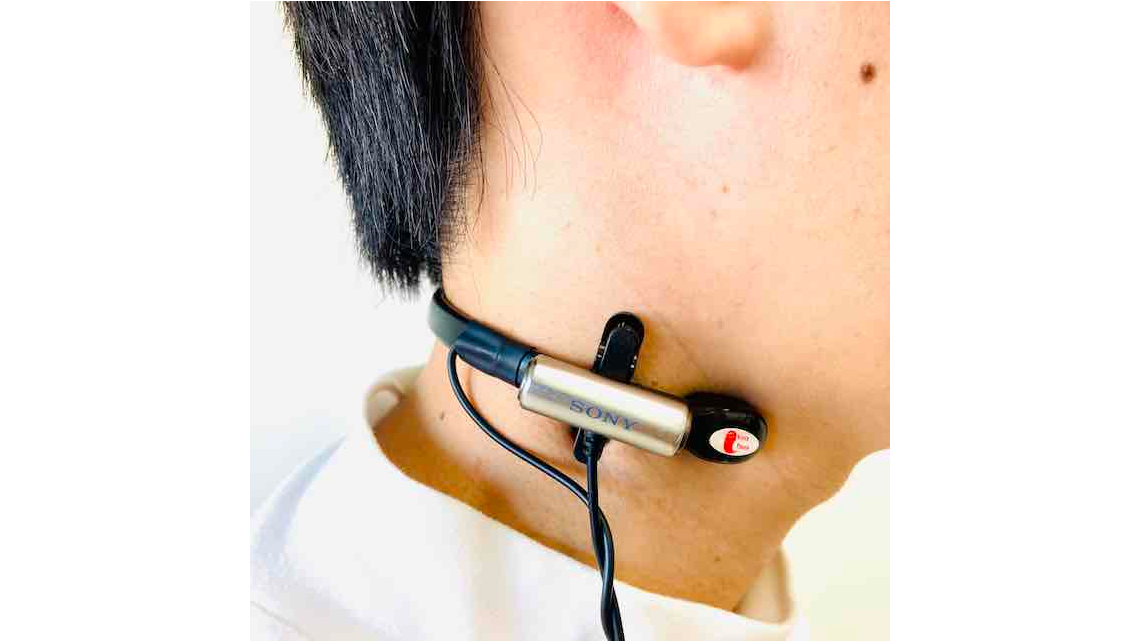
Throat and lavalier microphone.

To record the conversational data, we asked the psychologist to wear a neckband-type throat microphone and a sound-collecting microphone (Figure 2) around the neck during the cognitive function test. We asked the same of the participants during the cognitive function test and the everyday conversations with the humanoid robot (Linear PCM format/44.1 kHz/stereo). The sound-collecting microphones also record environmental noises and the voices of people in a location distant from the target speaker. For that reason, it becomes difficult to accurately estimate the target speaker's speech interval when there are speech superimpositions or loud environmental sounds. Therefore, we decided to add throat microphones that do not easily pick external environmental sounds [31].

### Feature Extraction

We extracted the speech features from the throat microphones and the sound-collecting microphones. Rather than comprehensively extracting many features using software, such as openSMILE [32], used in the previous study, we selected and extracted those features that may reflect the physiological changes in patients with MCI that could help doctors diagnose patients with MCI. Table 2 shows the extracted features. For each feature, we set the duration from the time when the psychologist or the robot started talking or asking a question to the time when the participants finished their replies as 1 turn and extracted features from each turn. In other words, since we extracted the response time and the silence time from every turn, we obtained as many values as the number of turns per participant.

**Table 2:** Prosodic and acoustic features.

Extracted feature	Description
Duration of response time (seconds)	Length of time between the beginning and the end of the participant's speech during a turn
Duration of reaction time (seconds)	Length of time between the end of the speech by the psychologist/the humanoid robot and the beginning of the participant's speech
Duration of speech periods (seconds)	Total value of speech periods in the duration of the response time
Duration of silent periods (seconds)	Total value of silent periods (300 ms or more) in the duration of the response time
Proportion of silent periods (%)	Percentage of the duration of silence periods in the duration of the speech period
Filler periods (seconds)	The total value of speech periods for fillers, such as “er,” in the duration of the speech period
Proportion of fillers (%)	The proportion of filler periods in the duration of the speech period
*F*_0cov_	Coefficient of variation of the fundamental frequency
Jitter (local, rap^a^, ppq5^b^, ddp^c^)	Fluctuations in pitch
Shimmer (local, apq3^d^, apq5, apq11, dda^e^)	Fluctuations in volume

^a^rap: relative average perturbation.

^b^ppq: period perturbation quotient.

^c^ddp: difference of difference of periods.

^d^apq: amplitude perturbation quotient.

^e^dda: average absolute differences between the amplitudes of consecutive periods.

#### Features Extracted From the Throat Microphones

We manually labeled speech intervals and fillers using the conversational data recorded with the throat microphone. Fillers are so-called pausing and conjunction words represented by speech forms, such as “er,” “um,” and “uh.”

The fillers in the conversation speech recorded in this study included not only “er” and “um” but also “well...” and “you see...” and the much rarer cases where participants dragged out the ending of the word, such as “Asawaaaa” (“in the morning”) and “XYZ wo tabete” (“I ate XYZ”), in order to buy time to think before continuing with the rest of the sentence. However, labeling this part of the speech as a filler would risk changing the meaning of the utterance as the semantic content from the word ”morning“ would be omitted. Therefore, we labeled it as an utterance. We conducted labeling on the principle that a part of an utterance is labeled as a filler only if the meaning of the utterance does not change even if it is removed.

We extracted 7 types of features concerning the speech intervals, such as the duration of the speech period and the proportion of fillers, using the speech interval and filler labels. The duration of the speech period, the response time, and the proportion of the silence time are features that previous studies have shown to be effective [12,15,17,18]. It is difficult to extract features such as the speech period and response time with sound-collecting microphones only when there are speech superimpositions. However, throat microphones enabled us to extract those more accurately.

#### Features Extracted From the Sound-Collecting Microphones

We extracted 10 types of acoustic features from the conversations recorded with the sound-collecting microphones. Although throat microphones help to filter out environmental noises, they are significantly different from usual acoustic microphones as they are skin conducting. Therefore, we decided to refer to the speech intervals obtained with the throat microphones and estimated the acoustic features using Praat [33] from the relevant section of the data collected with the sound-collecting microphones. We deleted the silent intervals and connected the sound segments to extract features.

Apart from local, jitter includes relative average perturbation (rap) and 5-point period perturbation quotient (ppq5). Similarly, shimmer also includes amplitude perturbation quotient (apq)3 and average absolute differences between the amplitudes of consecutive periods (dda), apart from local, that require different methods of calculation [34,35].

Since the basic frequency (*F*_0_) tends to be influenced by gender, we decided to use the coefficient of variation of *F*_0_ (*F*_0cov_) used by Tanaka et al [19]. The following equation shows the calculation of *F*_0cov_.

*F*_0cov_ = *F*_0_ SD/*F*_0_ mean

### Statistical Analysis

We conducted a statistical significance test for each feature to clarify the speech features that are useful when classifying people into CN and MCI groups. We checked for normality in advance with the Shapiro–Whisk test and found none in all the features. Moreover, when we checked for the homoscedasticity of each feature value using the *F* test, we confirmed it in 1 feature in the everyday conversations with the humanoid robot (proportion of silent periods) and 9 (*F*_0cov_, jitter [local, rap, ppq5, ddp], and shimmer [local, apq3, apq11, dda]) in the MMSE conversational speech. Therefore, we adopted the Brunner-Munzel test, which is nonparametric and usable when there is no assumption of homoscedasticity [36].

We defined a significant difference to be when *P*<.05. In addition, we used an effect size r to evaluate discriminability. The reference for the effect size was 0.10 (small), 0.30 (medium), and 0.50 (large) [37]. Table 3 shows the sample size of each experiment.

**Table 3:** Sample size of each experiment.

Experiment	CN^a^, n (%)	MCI^b^, n (%)
MMSE^c^ conversational speech	454 (49)	475 (51)
Speech in the everyday conversations with the humanoid robot	1070 (51)	1034 (49)

^a^CN: cognitively normal.

^b^MCI: mild cognitive impairment.

^c^MMSE: Mini-Mental State Examination.

### Automatic Classification Experiment

We conducted an experiment on the automatic classification of patients with MCI and CN older people using the features clarified in the statistical significance test to examine the effectiveness of simple screening tests for dementia through everyday conversations with humanoid robots. We used the following 4 types of features: significant feature plus age (at the time of the experiment), significant feature, “significant and effect size over 0.1” feature plus age, and “significant and effect size over 0.1” feature.

Since we extracted each feature at every turn (from the time the psychologist or Pepper asked a question until the participant finished their reply), we obtained as many features as the number of questions per participant. Therefore, we calculated arithmetic values (mean, variance, median, range) to convert these features into 1 feature value per participant. After that, we normalized the feature value to fall within the range of 0-1.

We used Waikato Environment for Knowledge Analysis (WEKA, University of Waikato, New Zealand) [38], an integrated software program for machine learning, for the automatic classification experiment. We conducted 5-fold cross-validations using the linear kernel of the SVM the previous studies had used for accuracy [13,20,21]. We used WEKA's Grid Search package for the tuning of cost parameters, which are hyperparameters.

#### Evaluation Indices

We used accuracy, sensitivity, and specificity as evaluation indices for the results. Accuracy refers to the percentage of correct classifications. We calculated the balanced accuracy in this study for performance evaluation to avoid the problem that accuracy is not suitable for a task when the result of the correct prediction of either positive or negative examples is extremely large or small. Balanced accuracy was used as an evaluation index. Sensitivity is an index of how correctly the experiment classified patients with MCI and the rate of not missing diseases. Specificity is an index of how correctly the experiment classified CN participants and avoided unnecessary doubts that CN older people have MCI. A result was true positive (TP) when a positive result was correct (ie, when the experiment classified the patients with MCI correctly) and false negative (FN) is when it classified them incorrectly. A result was false positive (FP) when the positive result was incorrect (ie, when the experiment mistook a CN older person as a patient with MCI) and true negative (TN) when it correctly classified a CN older person as being CN. The following equations show the calculation of balanced accuracy, sensitivity, and specificity:

Balanced accuracy = (Sensitivity + Specificity)/2

Sensitivity = TP/(TP + FN)

Specificity = TN/(FP + TN)

### Questionnaires on the Speech Dialogues With a Humanoid Robot

We administered questionnaires (Textbox 2) before and after recording the participants' everyday conversations with the humanoid robot to confirm the possibility of the participants accepting having conversations with robots as an everyday activity.

**Question contents.**

Questionnaire before recording (yes/no/don't remember):

- Have you ever had a conversation with a robot?

Questionnaire after recording (rating method: on a scale of 1-5, the closer to 5, the better):

- Did you find the conversation with the robot natural?
- Do you want to talk to a robot again?

## Results

### Results of the Statistical Significance Test

Table 4 shows the result of the statistical significance test of the features extracted from the MMSE conversational speech of CN older people and patients with MCI. Table 5 shows the result of the statistical significance test of the features extracted from the participants' speech in their everyday conversations with the humanoid robot.

We obtained significant differences in 5 (29%) of 17 types of features obtained from the MMSE conversational speech, of which 3 (60%) met the reference value of the effect sizes. In contrast, we obtained significant differences in 16 (94%) of 17 types of features obtained from the speech in the everyday conversations with the humanoid robot, of which 1 (6%) met the reference value of the effect sizes. We obtained significant differences in 11 more types of features obtained from the speech in the everyday conversation with the humanoid robot and 4 fewer features that met the reference value of the effect sizes compared to the MMSE conversational speech.

**Table 4:** Result of the statistical significance test of the features extracted from the MMSE^a^ conversational speech.

Features	CN^b^, mean (SD)	MCI^c^, mean (SD)	*P* value	Effect size r
Duration of response time (seconds)	2.19 (2.80)	3.08 (5.78)	.11	0.05
Duration of reaction time (seconds)	0.53 (1.20)	0.69 (1.05)	<.001	0.13
Duration of speech periods (seconds)	1.64 (1.55)	1.91 (2.58)	.74	0.01
Duration of silent periods (seconds)	0.42 (1.38)	0.95 (3.13)	<.001	0.13
Proportion of silent period (%)	0.06 (0.14)	0.11 (0.19)	<.001	0.13
Filler periods (seconds)	0.11 (0.36)	0.19 (0.50)	.01	0.09
Proportion of fillers (%)	0.04 (0.11)	0.05 (0.12)	.02	0.08
*F*_0cov_	0.25 (0.18)	0.24 (0.18)	.42	0.03
Jitter (local)	0.04 (0.01)	0.04 (0.01)	.49	0.02
Jitter (rap^d^)	0.02 (0.01)	0.02 (0.01)	.54	0.02
Jitter (ppq5^e^)	0.02 (0.01)	0.02 (0.01)	.42	0.03
Jitter (ddp^f^)	0.05 (0.02)	0.05 (0.02)	.54	0.02
Shimmer (local)	0.14 (0.04)	0.14 (0.04)	.30	0.03
Shimmer (apq3^g^)	0.06 (0.02)	0.06 (0.02)	.72	0.01
Shimmer (apq5)	0.09 (0.03)	0.09 (0.04)	.36	0.03
Shimmer (apq11)	0.15 (0.06)	0.15 (0.06)	.65	0.01
Shimmer (dda^h^)	0.19 (0.07)	0.19 (0.07)	.72	0.01

^a^MMSE: Mini-Mental State Examination.

^b^CN: cognitively normal.

^c^MCI: mild cognitive impairment.

^d^rap: relative average perturbation.

^e^ppq: period perturbation quotient.

^f^ddp: difference of difference of periods.

^g^apq: amplitude perturbation quotient.

^h^dda: average absolute differences between the amplitudes of consecutive periods.

**Table 5:** Result of the statistical significance test of the features extracted from the participants' speech in their everyday conversations with the humanoid robot.

Features	CN^a^, mean (SD)	MCI^b^, mean (SD)	*P* value	Effect size r
Duration of response time (seconds)	4.62 (7.33)	3.83 (5.05)	.02	0.05
Duration of reaction time (seconds)	0.90 (1.02)	1.15 (1.33)	<.001	0.08
Duration of speech periods (seconds)	3.39 (5.09)	2.62 (3.01)	<.001	0.09
Duration of silent periods (seconds)	1.29 (2.46)	1.12 (2.12)	.02	0.05
Proportion of silent period (%)	0.15 (0.21)	0.14 (0.22)	.13	0.03
Filler periods (seconds)	0.19 (0.60)	0.15 (0.54)	.01	0.06
Proportion of fillers (%)	0.03 (0.08)	0.02 (0.07)	.01	0.05
*F*_0cov_	0.21 (0.12)	0.20 (0.14)	<.001	0.09
Jitter (local)	0.029 (0.010)	0.031 (0.013)	<.001	0.10
Jitter (rap^c^)	0.013 (0.005)	0.014 (0.007)	<.001	0.09
Jitter (ppq5^d^)	0.014 (0.005)	0.016 (0.006)	<.001	0.09
Jitter (ddp^e^)	0.039 (0.01)	0.043 (0.02)	<.001	0.09
Shimmer (local)	0.12 (0.026)	0.13 (0.031)	<.001	0.09
Shimmer (apq3^f^)	0.05 (0.02)	0.06 (0.02)	<.001	0.08
Shimmer (apq5)	0.075 (0.019)	0.078 (0.024)	<.001	0.07
Shimmer (apq11)	0.119 (0.037)	0.123 (0.041)	<.001	0.06
Shimmer (dda^g^)	0.16 (0.05)	0.18 (0.06)	<.001	0.08

^a^CN: cognitively normal.

^b^MCI: mild cognitive impairment.

^c^rap: relative average perturbation.

^d^ppq: period perturbation quotient.

^e^ddp: difference of difference of periods.

^f^apq: amplitude perturbation quotient.

^g^dda: average absolute differences between the amplitudes of consecutive periods.

### Results of the Automatic Classification Experiments for Patients With MCI and Cognitively Normal Older People

#### Baseline

The cut-off value of the MMSE scores is used as a criterion for judging whether a person is suspected of having dementia when conducting the MMSE. We used existing knowledge and set the result of classification that used the value as the baseline. When the MMSE score is 27 points or less out of 30, it indicates the suspicion of MCI; 23 points or less indicate the suspicion of dementia [39]. In this study, we set 27 points as the threshold for dividing the participants into patients with MCI and CN older people. Table 6 shows the result of the classification using this threshold.

The classification using this threshold showed that it was possible to classify people into patients with MCI and CN older people with an accuracy of 53.2%. Sensitivity and specificity were generally consistent with the findings in the MMSE: a score of 27/28 proved to be most promising (sensitivity of 66.34% and specificity of 72.94%) in differentiating MCI and CN [39].

**Table 6:** Result of the classification using the score threshold.

	Accuracy (%)	Sensitivity (%)	Specificity (%)	Features, n
MCI^a^-CN^b^	53.2	40.4	66.0	1

^a^MCI: mild cognitive impairment.

^b^CN: cognitively normal.

#### Result of the Automatic Classification Experiment

Table 7 shows the result of the experiment of automatic classification of patients with MCI and CN older people using the MMSE conversational speech. Table 8 shows the result using everyday conversations with a humanoid robot. The result showed that the automatic classification exceeded a baseline accuracy of 53.2% with both the MMSE conversational speech and everyday conversations with the humanoid robot, which demonstrates the possibility of identifying patients with MCI using the features obtained in this study.

**Table 7:** Result of the experiment on automatic classification of patients with MCI^a^ and CN^b^ older people using the MMSE^c^ conversational speech.

Type of feature	Accuracy (%)	Sensitivity (%)	Specificity (%)	Features, n (%)
Significant feature plus age	62.8	55.3	70.2	21 (30)
Significant feature	61.7	57.4	66.0	20 (29)
“Significant and effect size over 0.1” feature plus age	63.8	66.0	61.7	13 (19)
“Significant and effect size over 0.1” feature	66.0	59.6	72.3	12 (18)

^a^MCI: mild cognitive impairment.

^b^CN: cognitively normal.

^c^MMSE: Mini-Mental State Examination.

**Table 8:** Result using everyday conversations with a humanoid robot.

Type of feature	Accuracy (%)	Sensitivity (%)	Specificity (%)	Features, n (%)
Significant feature plus age	66.0	61.7	70.2	65 (94)
Significant feature	57.4	48.9	66.0	64 (94)
“Significant and effect size over 0.1” feature plus age	68.1	70.2	66.0	5 (7)
“Significant and effect size over 0.1” feature	56.4	34.0	78.7	4 (6)

For the MMSE conversational speech, the result showed that it is possible to classify people into patients with MCI and CN older people with an accuracy of 66.0% (“significant and effect size over 0.1” feature).

Compared to the classification result based solely on MMSE scores, the result with the speech in everyday conversations with the humanoid robot showed that it is possible to classify people with an accuracy of 68.1% (14.9% higher; “significant and effect size over 0.1” feature plus age). In addition, the result showed that it is possible to identify patients with MCI from speech in everyday conversations with a humanoid robot with the same degree of accuracy as that of the automatic classification from the MMSE conversational speech. These results demonstrate that we can conduct a simple dementia-screening test using everyday conversations with a humanoid robot.

### Result of Questionnaires on Speech Dialogues With a Humanoid Robot

We received responses from 43 (91%) of the 47 CN older participants and 42 (89%) of the 47 patients with MCI. Of the 43 CN older participants, 6 (14%) and of the 42 patients with MCI, 4 (10%) replied that they had had a conversation with a robot before. Of the 85 participants who responded to both questionnaires, 75 (88%) had no previous experience of having a conversation with a robot and did it for the first time in this experiment.

**Figure 3:**
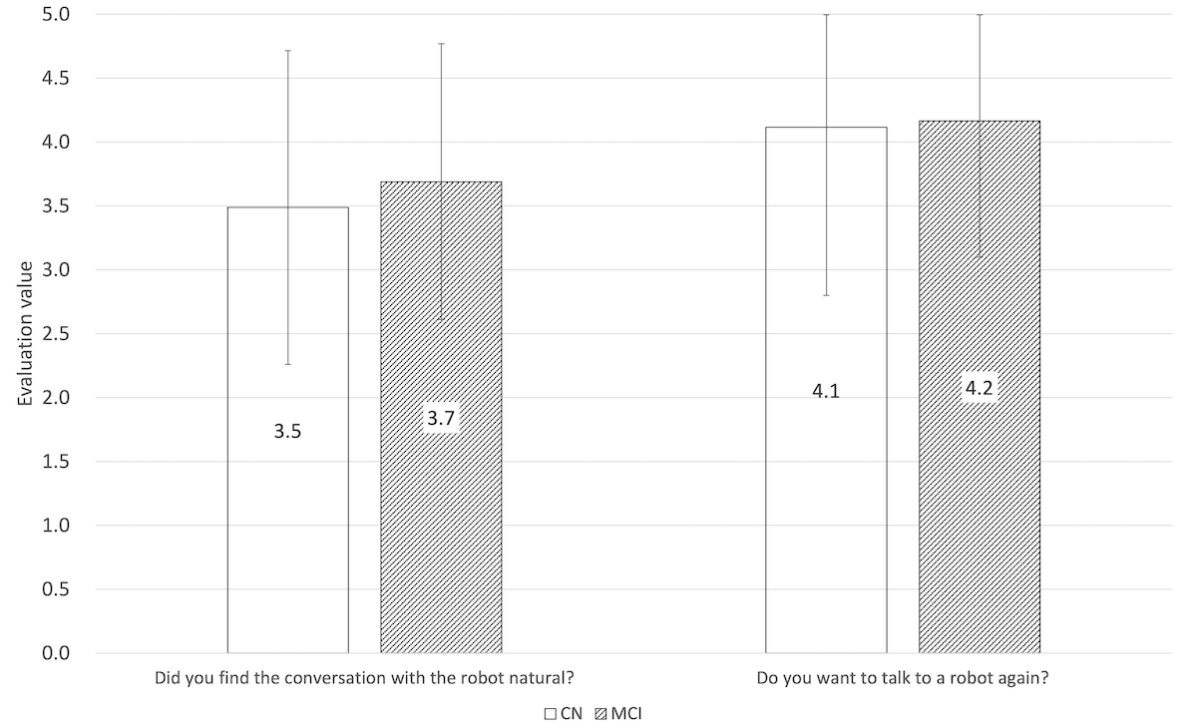
Result of questionnaires on the speech dialogues with the humanoid robot. CN: cognitively normal; MCI: mild cognitive impairment.

The questionnaire administered after the conversation with the humanoid robot had a 0-5 rating scale. The closer the rating to 5, the more the participants felt the conversations with the robot were natural and wanted to talk with it again in the future. Figure 3 shows the result.

## Discussion

### Principal Findings

We analyzed speech features from the MMSE conversational speech and everyday conversations with a humanoid robot to identify salient features for detecting signs of cognitive decline. Furthermore, we conducted an automatic classification experiment with patients with MCI and CN older people using the features that were identified using a statistical significance test and examined the possibility of a simple dementia-screening test using everyday conversations with humanoid robots. We garnered significant differences in 11 more features in the everyday conversations with the humanoid robot than those found in the MMSE conversational speech. We also identified significant differences in acoustic features, such as jitter and shimmer, which were not present in the MMSE conversational speech. The results of automatic classification showed 66.0% accuracy for the MMSE conversational speech and 68.1% accuracy for everyday conversations with a humanoid robot.

This result showed that it is possible to identify patients with MCI from the speech in everyday conversations with a humanoid robot with the same degree of accuracy as that of the automatic classification from the MMSE conversational speech. Furthermore, on the question of whether the participants want to talk with a robot again, both CN older participants and patients with MCI scored the responses higher than 4 points. We concluded that the participants are positive about having future everyday conversations with robots again.

The response and speech times of patients with MCI tended to be more attenuated than those of CN older people in the MMSE conversational speech. Conversely, we noted an opposite trend, where their response and speech times were less attenuated in everyday conversations with the humanoid robot. Language and communication difficulties are known to be common symptoms in people with dementia [40,41]. Unlike cognitive function tests, such as the MMSE, real everyday conversations do not have fixed answers, and it was up to the participants themselves to decide how much they would talk. The patients with MCI whose cognitive functions were beginning to decline may have found it more difficult to communicate with the humanoid robot than the CN older participants did. This may be why we found the opposite trend where the response and speech times of the patients with MCI were longer in the MMSE conversational speech, even though they were shorter in the everyday conversations with the humanoid robot. We found significant differences in the feature values of vocal fluctuations, such as jitter and shimmer, in everyday conversations with the humanoid robot, unlike the MMSE conversational speech. In the MMSE conversational speech, this may be because it was difficult for vocal fluctuations to occur in speech since the participants could provide short replies in the tasks of the orientations. Conversely, it seems that vocal tremors occurred due to the nature of the everyday conversation. A comparison of the average value shows that it was higher for the patients with MCI than for the CN older participants, which means that members of the former group tend to have a more fluctuating voice in terms of pitch and volume. This showed that jitter and shimmer in everyday conversations with a humanoid robot are effective markers for detecting signs of cognitive decline.

In automatic classification, the specificity (the index of avoiding an unnecessary suspicion of dementia) tends to be higher than sensitivity (the index of identifying patients with MCI without fail) except for the “significance and effect size over 0.1” feature. Conversations with a humanoid robot simulate those that take place between people and are not created specifically to identify patients with MCI. This may explain why specificity tends to be higher. In terms of identifying patients with MCI without fail, the relevant index is sensitivity, which was higher compared to the other features for both the MMSE conversational speech and conversations with the humanoid robot when we used features at the “significant and effect size over 0.1” feature plus age. It seems that we can improve the accuracy of the performance (identifying patients with MCI without fail) by narrowing it down to a feature that meets the reference value of the effect size. However, sensitivity decreased significantly to 34.0% compared to the other values in the automatic classification experiment using the “significance and effect size over 0.1” feature. This indicates that age may have a great deal of influence. Although age is considered a significant factor in cognitive decline, it is unlikely to reflect the changes caused by cognitive decline, as age increases every year. Although it may be effective to use features that met the reference value of the effect size, further examinations are needed to determine whether to add age to the features.

In addition, on the question of whether the conversation with the robot was natural, the rating of the patients with MCI was 0.2 points higher than that of the CN older participants. Patients with MCI may not feel awkward or uncomfortable talking with a robot, since they have reduced cognitive function compared to CN older people. This may be why the patients with MCI found the conversation with the robot more natural than the CN older people did.

The problem with the everyday conversations with the humanoid robot was that the classification accuracy was up to 68.1%, which is an issue in terms of early detection of dementia. It seems that the low accuracy is caused by using only acoustic features and those acoustic features for which statistically significant differences have been confirmed. It is necessary for improving accuracy for practical use. However, accuracy increased by 14.9% when we classified the participants solely with the MMSE score set as the baseline. We may reduce the risk of not identifying patients with MCI by also using speech in everyday conversations with a humanoid robot, rather than simply judging the performance from the MMSE test scores alone. In the future, we will examine how to improve the accuracy of automatic classification from everyday conversations with a humanoid robot by analyzing speech content and new features, such as linguistic features, in the practical use of robots.

This study successfully demonstrated that it is possible to classify people into patients with MCI and CN older people even from somewhat unnatural conversations with a humanoid robot with the same level of accuracy as found with human conversations in MMSE tests. This means the study has shown that humanoid robots can identify signs of cognitive decline while having everyday conversations with people in nursing homes and other similar facilities where labor shortages are an acute problem.

### Conclusion

This study examined a simple screening test for dementia that uses everyday conversations with a humanoid robot installed at nursing homes and similar facilities. Existing studies have not clarified what the effective features are for identifying patients with MCI, a precursor stage of dementia, from the speech in conversations with a humanoid robot. Therefore, we recorded speech in conversations between the participants of the experiment (CN older people and patients with MCI) and a humanoid robot, extracted 17 types of features from the recordings, and conducted a statistical significance test. From the result, we obtained significant differences between the CN older participants and the patients with MCI in 16 types of features, such as response time, speech time, jitter, and shimmer, and clarified effective features for detecting signs of cognitive decline through conversations with a humanoid robot.

We conducted an automatic classification experiment using an SVM to verify whether it is possible to automatically classify people into patients with MCI and CN older people from their speech in everyday conversations with a humanoid robot by examining the features identified in this study. The results showed that we can classify people into patients with MCI and CN older people with an accuracy of 68.1% from their everyday conversations with a humanoid robot. The accuracy increased by 14.9% compared to the classification conducted solely with the MMSE score (a cognitive test) we had set as the baseline.

These results suggest that we can identify patients with MCI from their everyday conversations with a humanoid robot and improve the efficacy of a simple screening test for dementia. However, the classification accuracy of the method is 68.1%, which is insufficient when we consider its prospective potential for practical use. Our future task is to improve its accuracy further by examining additional features, such as those of a linguistic nature.

## Abbreviations

apq: amplitude perturbation quotient
CN: cognitively normal
dda: average absolute differences between the amplitudes of consecutive periods
ddp: difference of difference of periods
FN: false negative
FP: false positive
HDS-R: Hasegawa method simple intelligence assessment scale
MCI: mild cognitive impairment
MMSE: Mini-Mental State Examination
ppq: period perturbation quotient
rap: relative average perturbation
SVM: support vector machine
TN: true negative
TP: true positive
WoZ: Wizard of Oz

## Data Availability

The data sets presented in this paper are not readily available but are derived, and supporting data may be available from the corresponding authors on reasonable request and with permission from the Ethics Committee, University of Tsukuba Hospital.
